# Radiomics and 256-slice-dual-energy CT in the automated diagnosis of mild acute pancreatitis: the innovation of formal methods and high-resolution CT

**DOI:** 10.1007/s11547-024-01878-9

**Published:** 2024-08-30

**Authors:** Aldo Rocca, Maria Chiara Brunese, Antonella Santone, Giulia Varriano, Luca Viganò, Corrado Caiazzo, Gianfranco Vallone, Luca Brunese, Luigia Romano, Marco Di Serafino, R O I Segmentation Collaborative Group, Fabio Bellifemine, Francesca De Chiara, Dalila De Lucia, Giulia Pacella, Pasquale Avella

**Affiliations:** 1 Department of Medicine and Health Science “V. Tiberio”, University of Molise, Campobasso, Italy; 2 Hepatobiliary Unit, Department of Minimally Invasive General and Oncologic Surgery, Humanitas Gavazzeni University Hospital, Bergamo, Italy; 3 Department of General and Emergency Radiology, AORN “Antonio Cardarelli”, Naples, Italy; 4 Department of Precision Medicine, University of Campania “L. Vanvitelli”, Naples, Italy

**Keywords:** Pancreatitis, Diagnosis, Radiomics, Artificial intelligence, Formal methods, Mild acute pancreatitis

## Abstract

**Introduction:**

Acute pancreatitis (AP) is a common disease, and several scores aim to assess its prognosis. Our study aims to automatically recognize mild AP from computed tomography (CT) images in patients with acute abdominal pain but uncertain diagnosis from clinical and serological data through Radiomic model based on formal methods (FMs).

**Methods:**

We retrospectively reviewed the CT scans acquired with Dual Source 256-slice CT scanner (Somatom Definition Flash; Siemens Healthineers, Erlangen, Germany) of 80 patients admitted to the radiology unit of Antonio Cardarelli hospital (Naples) with acute abdominal pain. Patients were divided into 2 groups: 40 underwent showed a healthy pancreatic gland, and 40 affected by four different grades (CTSI 0, 1, 2, 3) of mild pancreatitis at CT without clear clinical presentation or biochemical findings.

Segmentation was manually performed. Radiologists identified 6 patients with a high expression of diseases (CTSI 3) to formulate a formal property (Rule) to detect AP in the testing set automatically. Once the rule was formulated, and Model Checker classified 70 patients into “healthy” or “unhealthy”.

**Results:**

The model achieved: accuracy 81%, precision 78% and recall 81%. Combining FMs results with radiologists agreement, and applying the mode in clinical practice, the global accuracy would have been 100%.

**Conclusions:**

Our model was reliable to automatically detect mild AP at primary diagnosis even in uncertain presentation and it will be tested prospectively in clinical practice.

## Introduction

Acute pancreatitis (AP) is a common gastrointestinal disease with an annual incidence rate of about 33.74/100 000, along with a yearly mortality rate of about 1.16/100 000 [1].

The incidence of AP has increased in the last few years, as it is strictly related to the diffusion of risk factors in the general population, such as alcoholism, obesity, type 2 diabetes, and cardiovascular and renal diseases [2].

Diagnosing acute pancreatitis requires at least 2 criteria among the evidence of signs at imaging, the increment of amylase or lipase of 3 times the normal value, and the clinical abdominal pain referred [3, 4]. Once the diagnosis has been formulated, AP must currently be classified into three degrees of severity: mild acute pancreatitis (MAP), moderately severe acute pancreatitis (MSAP), and severe acute pancreatitis (SAP) [5].

Most AP are treated conservatively, and only the 15% evolve into severe acute pancreatitis, but in order to early identify the high-risk patients,

several scores have been proposed to determine AP severity. The acute physiology and chronic health evaluation II (APACHE II) score has the best accuracy, even if it collects only clinical parameters [6]. On the other hand, the computed tomography (CT) severity index (CTSI) and modified CTSI (mCTSI), instead, show a comparable prognostic performance including CT imaging data [6].

While the diagnosis of severe acute pancreatitis, is determined by evident signs on CT images, as large areas of necrosis in the retroperitoneum and fluid film in the hepatorenal and splenorenal spaces, the diagnosis of mild acute pancreatitis can still be challenging considering the minimal radiological features detectable at standard CT scan protocols [7]. Nonetheless, an early diagnosis is crucial especially considering its unpredictable evolution into a benign or severe clinical picture a few days later.

Nowadays the mean accuracy of radiologists in the identification of AP is about 94%, but, it decreases to 80% in the identification of mild pancreatitis [7]. The accuracy can be limited by the absence or a low evidence of pathognomonic signs such as typical fluid collection and gland edema (CTSI score = 0) [7].

These features can become evident on CT images in a later time, and when it is too late to change clinical management, with consequent development of necrosis. 30% of these patients will develop a superimposed necrosis infection, and consequent poor prognosis [8].

To overcome these limitations the latest advances in CT technology aim to ensure higher resolution with the lowest-dose exposure. The diagnostic performance has already been tested on pancreatic tumors [9, 10].

The utility of Radiomics has already been demonstrated in many fields to better define the evolution of clinical pictures through radiological data [11–15].

Radiomics is the study of quantitative features of radiologic imaging using, for example, texture analysis and has been shown to provide additional information on underlying pathology in many diseases as in the oncological field and medical or surgical emergencies [16–18].

The application of Radiomics on medical images leads to a large amount of data that can be analyzed effectively with artificial intelligence (AI) [19, 20].

Unfortunately, the imposing use of deep learning in these cases has disadvantages, such as the lack of explainability of the classification process and the need for large datasets, which is not always possible in the case of rare diseases or health emergencies [21, 22].

This is why there is a demand for valid alternatives, such as formal methods [23]: mathematical methods for verifying the behavior of complex systems. Such methods are used in Software Security to check the correct functioning of critical systems involving human lives and money movements [24].

Formal Methods have already demonstrated good reliability and versatility in many fields, such as oncological disease and COVID-19.

They have the great advantage of ensuring reliability with a small sample size, as they do not need the training and the validation set of machine learning methodology. Being mathematical techniques, Formal Methods are also able to be explicable in the classification process since the steps taken by the processor in making the final decision can be retracted [25, 26].

This research aims to explore the potentialities of Radiomics through Formal Methods, for the first time in the large field of emergency radiology. In particular, we aim to achieve automated diagnosis of acute mild pancreatitis looking for additional features not visible at the “naked eye view”, in order to improve the diagnostic performance of CT imaging.

### Primary endpoint of the study

To evaluate the performance of an automated tool to diagnose mild pancreatitis according to the radiomic characteristics of the pancreas. To compare the diagnostic performance of an expert radiologist supervising with that of the automated tool. Radiological diagnosis of mild pancreatitis was based on CTSI criteria [6].

## Material and methods

### Dataset

The study was conducted according to the guidelines of the Declaration of Helsinki and approved by the Institutional Review Board of the University of Molise (protocol number 20/23, approval date: Jul 05, 2023).

We retrospectively collected contrast-enhanced CT scans of 80 consecutive compliant patients admitted to the Radiology Unit of Antonio Cardarelli Hospital of Naples in 2022, showing acute abdominal pain and a suspect of acute pancreatitis.

The reference for the standard was the diagnosis of mild pancreatitis according to international definitions, i.e., combining clinical, laboratory and radiological data [27].

The inclusion criteria were:

- age > 18 years old;
- CT images acquired with a standard predefined acquisition protocol

The exclusion criteria were:

- evidence of focal or diffuse benign or malignant pancreatic lesions;
- diagnosis of severe acute pancreatitis, chronic pancreatitis, and severe involution of the pancreatic gland;
- lack of compliance to be part of the study.

80 patients were divided into two groups.

The first group is composed of 40 patients who underwent biphasic contrast-enhanced (CE)-CT for acute abdominal pain but showing a pancreatic gland without CT signs of inflammation.

The second group is composed of 40 patients with acute abdominal pain affected by mild pancreatitis, diagnosed with biphasic CE-CT imaging or later through clinical presentation or laboratory findings.

The CT images were reviewed by 3 blind radiologists and supervised by one blind 20 year experience radiologist.

### Image acquisition

All patients underwent examination using a Dual Source 256-slice CT scanner (Somatom Definition Flash; Siemens Healthineers, Erlangen, Germany) with a craniocaudal scan of the abdomen in the supine position with abducted upper limbs. Additionally, a breath-hold technique was employed to prevent motion artifacts.

The contrast-enhanced CT (CE-CT) protocols consisted of two phases that were obtained following the injection of contrast media (CM) via intravenous (IV) administration. These phases were timed with specific scan times and included a virtual non-contrast (VNC) image. The CT parameters are reported in Table 1.

**Table 1:** CT acquisition protocol

CT acquisition parameters		Contrast-enhanced protocol	
Collimation	64 × 0.6 mm	High concentration of IV CM (Iomeprol 400 mg I/mL, Iomeron 400; Bracco, Milan, Italy)	1.2 ml per kilogram of body weight
Spatial resolution	0.30 mm		
Scan time	12 s		
Scan length	287 mm	Flow rate	3.5–4 mL/s
Rotation time	0.33 s	ROI	150 HU
Tube settings	100/140 kV, 50/60 mAs	Arterial phase delay	15 s
Effective dose	2.6 mSv	Portal phase delay	70–80 s

In most cases, an additional late scan of the abdomen at 3–5 min was not necessary, except in situations with inconclusive findings.

The virtual non-contrast (VNC) image was obtained by subtracting the iodine-enhanced images, reducing the overall radiation exposure to the patient. The scanned volume included the abdominal area from the diaphragm to the pubic branches, with an effective slice thickness of 2.5 mm and reconstruction thickness set at 0.625 mm to facilitate thorough analysis and post-processing reformatting.

### Segmentation and features extraction

We included venous CE- CT images with a slice thickness of 2.5 mm.

**Fig. 1:**
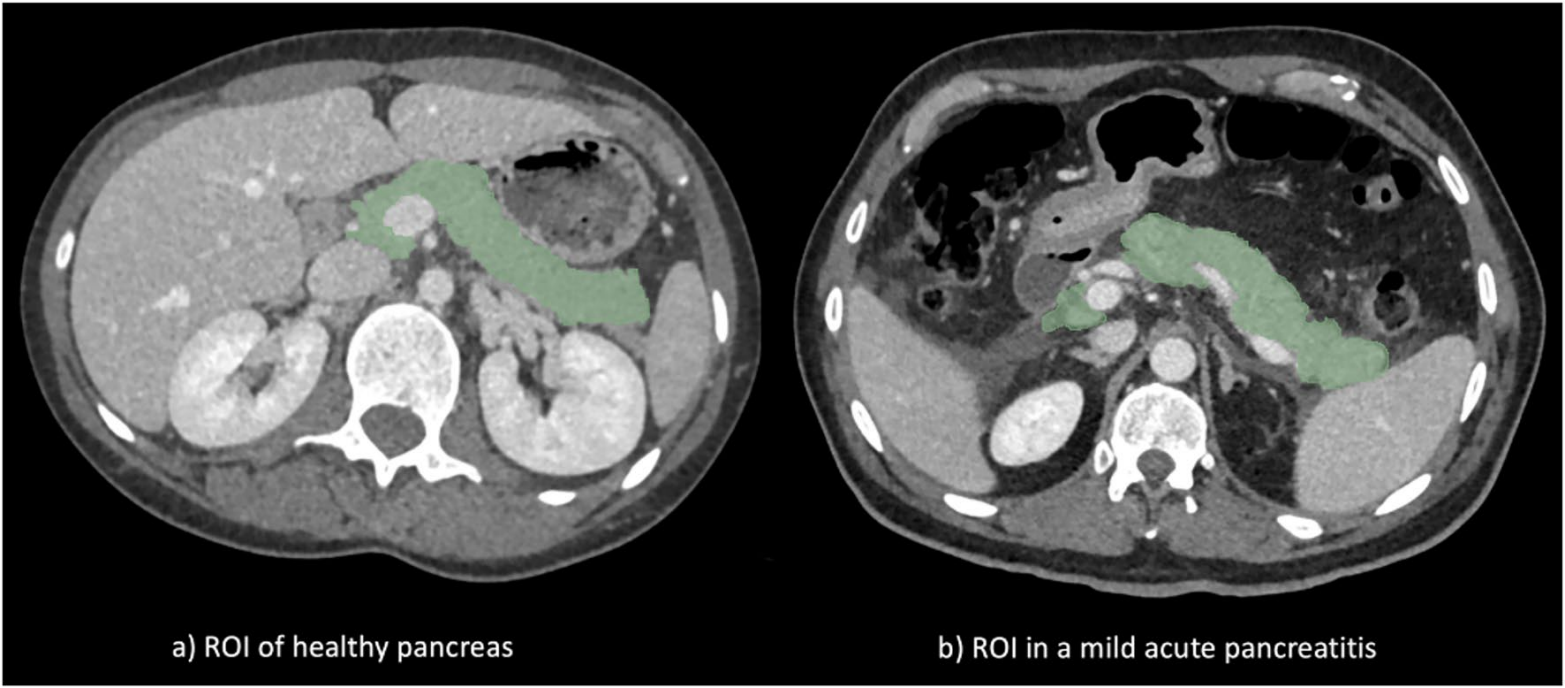
**a** ROI of healthy pancreas patient; **b** ROI in a mild acute pancreatitis patient automatically detected

Region of interest (ROI) has been manually defined by 6 different operators slice by slice for each patient. Before starting manual segmentation, a protocol has been defined to avoid high inter-operator variability (Fig. 1) The ROI included the pancreatic gland, rigorously excluding the major vessels (portal vein, mesenteric vein, splenic vein), the duodenum, and the small bowel. Whenever a peripancreatic fluid was present, it was included in the segmentation.

Healthy patients had a mean number of 19, 7 ROI (6–27 95% CI); while, unhealthy patients had a mean number of 22, 7 ROI (19–31 95% CI).

Successively, radiomic features were extracted through “PyRadiomics” (Boston, Massachusetts, USA) [28].

PyRadiomics is an extension of the 3D Slicer software, representing a Python library that allows the operators to convert the radiological images uploaded in DICOM format into numerical data, translating images into features, and so into numbers [29].

### Features selection

The 107 features extracted through PyRadiomics were visualized and then selected through Weka (General Public License, New Zealand), which is open-source software that, through machine learning algorithms and data mining, is able to identify the most discriminating features on a CSV dataset.

In this way, AI can predict new data behaviors. In the present study, Weka selected the most discriminating features related to “healthy” and “unhealthy” pancreas.

The further step consists in a feature reduction or selection [30]. The features reduction outputs resulted into 6 classes of features reported in Table 2.

**Table 2:** 6 classes of featured extracted

FIRST	GLCM	GLDM
10Percentile	Imc2	Dependence entropy
Mean	Joint entropy	Low gray level emphasis
Median	Sum entropy	Dependence non uniformity
Skewness	Maximum probability	
Uniformity		
GLSZM	SHAPE	GLRLM
Zone Entropy	Maximum 2D Diameter Row	Gray level non uniformity
Small area high gray level emphasis	Minor axis length	Low gray level run emphasis
Size zone non uniformity	Surface area	Run entropy
		Short run low gray level emphasis

### Formal methodology

Once the most suitable features for the case study have been available, formal models are created by discretizing the values and translating them into a mathematical syntax called “CCS”.

The intervals are five and equivalent, called *‘very low’*, *‘low’, ‘basal’, ‘high’* and *‘very high’*: their presence or absence in the patient’s medical image series is fundamental for classification. In this mathematical technique there is no need for a testing and training set, therefore at the time of classification there is a need for knowledge extraction by a domain expert, e.g., the radiologist.

In order to differentiate pancreatitis from healthy pancreas, two experienced radiologists selected 6 patients typically affected by MAP (CTSI score 3) to draw up a formal property (or rule) to perform the classification task. These patients were chosen from our database considering the high expression of edema, gland density or involution, and the peripancreatic fluid.

The rules are defined to discriminate the presence of radiological features of MAP, so there is no need to define rules from healthy patients, who are necessary only as control validation group.

**Fig. 2:**
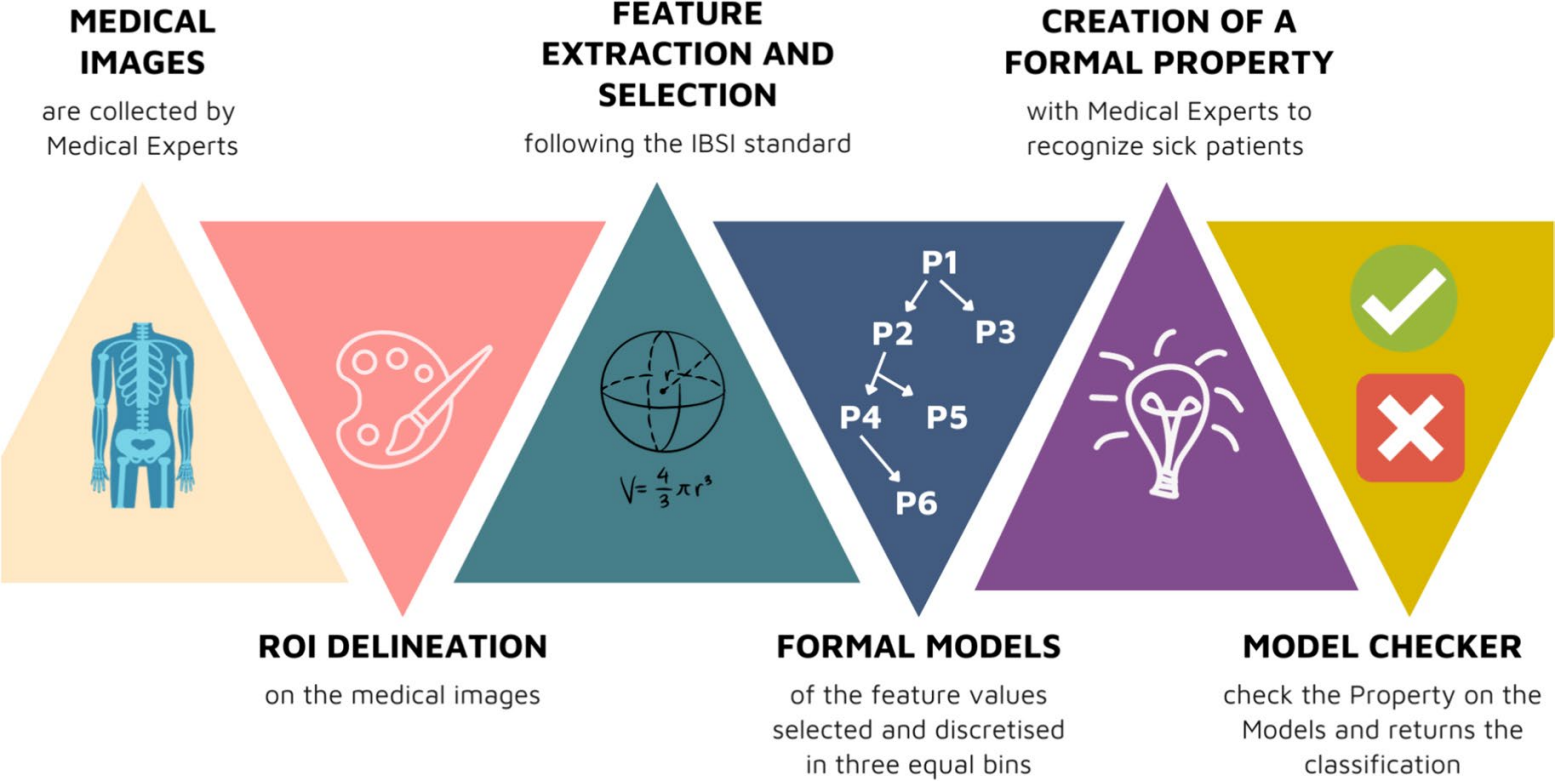
Schema of the methodology: starting from the medical images, there is the ROI segmentation step defining the pancreas region. After feature extraction and selection, the mathematical models are created and then verified through the formal Property written by radiologists and computer scientists. The Model Checker checks if the Property is satisfied or not, indicating the patient as healthy or affected by pancreatitis

The Property has been verified by the model checker, a mathematical agent checking if the Property is satisfied or not by the patients, on the residual cohort of patients (Fig. 2) [31–33].

## Results

During the analysis, the features extracted from 4 patients (one healthy, and three with MAP) resulted in incomplete; therefore, the patients were excluded from the cohort. Overall, 76 patients were considered, including 39 healthy subjects (controls), and 37 patients with MAP (cases). The latter group included 6 patients (with CTSI of 3) selected to draw up the Formal Property rule.

The most performing rule was obtained from 3 of the 6 patients, previously considered by the radiologists, it was defined from the feature classes FIRST and SHAPE selected from the following patients **FIRS CSV_20_CSV35 OR SHAP_CSV20_CSV29.**

Then the rule was tested on 70 patients. The results are reported in Table 3. 32 healthy patients were correctly classified as negative by formal methods.

**Table 3:** Results of the formal model checker in the testing dataset (*n* = 70); TP = true positive, FP = false positive; FN = false; negative; TN = true negative

Confusion matrix	Actual values	
Predicted values	TP 25	FP 7
	FN 6	TN 32
Diagnostic performance *n* = 70	**Accuracy**	0,81
	**Precision**	0,78
	**Sensitivity**	0,81
	**Specificity**	0,84

81% of patients affected by MAP were recognized by the radiomics model alone.

**Fig. 3:**
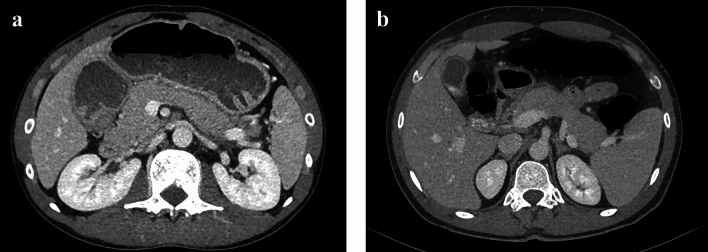
**a** An example of a young patient **not** affected by mild acute pancreatitis misdiagnosed by some of the radiologists and correctly classified by FMs, **b** An example of a young patient affected by mild acute pancreatitis misdiagnosed by some of the radiologists and correctly classified by FMs

Figure 3a and b shows the CT scans of some patients misdiagnosed by radiologists and correctly classified by AI.

Blind radiologists also viewed the exams and their inter-operator agreement was verified.

6 patients affected by MAP with a CTSI score of 0–1 were missed by some of the radiologists, but only 2 of these 6 patients were also missed by the Radiomic model.

2 young healthy patients were overdiagnosed with MAP by the radiologists. Both the 2 patients were correctly classified by Formal Methods.

The diagnostic performance achieved by the combined radiomics and radiologists model is reported in Table 4.

**Table 4:** Diagnostic performance of the combined model radiologists and formal methods

Confusion matrix	Actual values	
Predicted values	TP 29	FP 0
	FN 2	TN 39
Diagnostic performance n = 70	**Accuracy**	0,97
	**Precision**	1,00
	**Sensitivity**	0,94
	**Specificity**	0,95

ROIs have been defined by 6 different operators. This process did not condition the feature extraction, feature selection and the formal verification.

## Discussion

Our study demonstrated how Formal Methods are reliable in recognizing in a short work time a mild acute pancreatitis without any clinical data or human job after the definition of the methodology. In particular, they achieved a great recall (or sensitivity) to detect MAP, as they achieved the best results in the identification of MAP in patients with CTSI 0 or 1. The model achieved great accuracy in the differentiation of both groups of patients. In particular, the healthy patients misclassified also by the radiologists as affected by pancreatitis were young (age < 40), normal or overweight, showing a hypertrophic gland, in a small retroperitoneal space, with a low percentage of bordering fat. Furthermore, also unhealthy misclassified patients had a different gland density due to the fibrotic involution. In fact, the only patients that presented these different features were the ones considered by the model as false negatives, but correctly classified by radiologists.

In our opinion the reliability of the model may give radiologists and clinicians the possibility of consulting the model in clinical practice. The global accuracy, in fact, is comparable with radiologists but the global accuracy of the model combined with the expertise of radiologists could achieve an accuracy of almost 100% in recognizing both the CTSI scores group 0–1 and 2–3 [7].

Furthermore, in clinical practice, early diagnosis must be the goal for clinicians and radiologists, as it allows an early prognostication and severity assessment of AP, crucial to improve patients prognosis and reduce mortality. However, the CTSI, which is based on CT images, only can predict prognosis at a later time (72 h) when the pancreatitis features are evident on CT. Our model can identify the earliest sign on the CT images to assess a severity radiomic score [27, 34].

To our knowledge, this is also the first study based on an algorithm created starting from the features extracted by 6 patients, with typical imaging of AP, selected and chosen by the agreement of two expert radiologists during the evaluation of radiological features. This innovation in our methodology gives our hybrid model a great explainability as the diagnostic rule has not been based only on the “naked-eye-view” features, but on a translation of the radiological features into numerical ones [35–37].

But another innovation of Formal methodology, despite the most common machine learning methodologies, consists in overcoming the limits of huge sample size and an accurate training set [38, 39].

Machine learning (ML) models are structured on a training set, a validation set and a testing set, as the software learns data from radiological images. Images can often be related also to clinical data inserted into the learning process of the model [40–42].

For this reason, our model has been developed without a ML training cohort. The Rule was manually defined on 3 of the 6 patients affected by mild acute pancreatitis, considered the most typical radiological picture of the disease by two expert radiologists.

Sparing the training set, the other patients segmented were used only to verify the rule (training/testing 6/70) and it was able to correctly classify the 81% of patients.

Furthermore, formal methods appear also reliable on a dataset of images, where ROIs have been manually defined by 6 different operators. This process did not condition the feature extraction and the results; therefore, the segmentation can be considered operator-independent.

So, besides other applications of Formal Methods in clinical practice, the application on AP shows different opportunities for the application of AI when compared to previous studies [43–45]. In our opinion, the chance to classify patients whose segmentation was performed manually by different operators represents one of the main goals of our study.

To our knowledge, this is also the first study concerning the automated diagnosis of acute pancreatitis, analyzing only the portal phase, as it reduces the segmentation work time, preserving the best enhancement of the pancreatic gland [46, 47].

It is also a novel study among the different applications of radiomics to the latest advances in technology in CT scans [48–50].

Radiomics studies applied to emergency medicine focused on the prognosis prediction, the identification and diagnosis of chronic pancreatitis, the differential diagnosis from pancreatic adenocarcinoma and autoimmune pancreatitis [51]. Even if several studies reported a methodology applied to a large sample of patients, they often did not have a healthy control group, but they focused only on patients affected by acute pancreatitis to estimate prognosis [52–54].

The retrospective nature of the imaging collection represents the main limitation of our study. The sample size of our cohorts is still limited, but they just represented a testing set for the rule that was still trained and validated. We also excluded chronic pancreatitis from our dataset, even if it could be a cause of acute abdominal pain during re-activation [55, 56]. Our model is reliable with a testing set representing 92% of patients.

This is a first step to verify the reliability of our hybrid methodology created by radiologists, clinicians, and informatics, but also to automatize the diagnosis of AP. The main goal of the study concerns the hospital care of patients with a high risk of acute pancreatitis, to avoid further complications. Infected acute necrotizing pancreatitis is an always very difficult clinical picture to manage due to the complex inflammatory reaction of the patients, so interventional therapies may be needed to solve sepsis. In particular, severe necrotic pancreatitis may require an interventional or surgery approach from skilled specialists. Despite minimally invasive approaches, in particular with robotic techniques have been demonstrated safe and effective also in gastric and HPB surgery, concerning pancreatitis [57–60] the current trend in the literature leans toward favoring endoscopic drainage and endoscopic necrosectomy when possible [61].

However, the best management for patients affected by ANP is still debated and should be discussed case by case to face with very dangerous and various clinical conditions, and may be different in referral and peripheral centers [61, 62].

Future studies will focus on prognosis prediction after the automatic identification of mild acute pancreatitis. The model will also be tested on a prospective cohort in an observational study.

## Conclusion

The present study demonstrated that radiomic data in a Formal Property model may lead to an automated and early diagnosis of MAP with adequate reliability, but a really strong accuracy if supervised by radiologists.

## Data Availability

The study was conducted according to the guidelines of the Declaration of Helsinki and approved by the Institutional Review Board of the University of Molise (Prot.n.19/2023).

## Abbreviations

AP: Acute pancreatitis
MAP: Mild acute pancreatitis
MSAP: Moderately severe acute pancreatitis
SAP: Severe acute pancreatitis
APACHE II: Acute physiology and chronic health evaluation II
CRP: C-reactive protein levels
CT: Computed tomography
CTSI: Computed tomography severity index
mCTSI: Modified CTSI
HAPS: Harmless acute pancreatitis score
BISAP: Bedside index for severity in acute pancreatitis
AI: Artificial intelligence
CE: Contrast enhanced
CM: Contrast media
VNC: Virtual non-contrast image
ROI: Region of interest
HU: Hounsfield unit
ML: Machine learning
